# Regiospecific synthesis of prenylated flavonoids by a prenyltransferase cloned from *Fusarium oxysporum*

**DOI:** 10.1038/srep24819

**Published:** 2016-04-21

**Authors:** Xiaoman Yang, Jiali Yang, Yueming Jiang, Hongshun Yang, Ze Yun, Weiliang Rong, Bao Yang

**Affiliations:** 1 Key Laboratory of Plant Resources Conservation and Sustainable Utilization, Guangdong Provincial Key Laboratory of Applied Botany, South China Botanical Garden, Chinese Academy of Sciences, Guangzhou 510650, China; 2 University of Chinese Academy of Sciences, Beijing, 100049, China; 3 Food Science and Technology Programme, c/o Department of Chemistry, National University of Singapore, 3 Science Drive 3, 117543 Singapore, Singapore

## Abstract

Due to their impressive pharmaceutical activities and safety, prenylated flavonoids have a high potent to be applied as medicines and nutraceuticals. Biocatalysis is an effective technique to synthesize prenylated flavonoids. The major concern of this technique is that the microbe-derived prenyltransferases usually have poor regiospecificity and generate multiple prenylated products. In this work, a highly regiospecific prenyltransferase (FoPT1) was found from *Fusarium oxysporum*. It could recognize apigenin, naringenin, genistein, dihydrogenistein, kampferol, luteolin and hesperetin as substrates, and only 6-*C*-prenylated flavonoids were detected as the products. The catalytic efficiency of FoPT1 on flavonoids was in a decreasing order with hesperetin >naringenin >apigenin >genistein >luteolin >dihydrogenistein >kaempferol. Chalcones, flavanols and stilbenes were not active when acting as the substrates. 5,7-Dihydroxy and 4-carbonyl groups of flavonid were required for the catalysis. 2,3-Alkenyl was beneficial to the catalysis whereas 3-hydroxy impaired the prenylation reaction. Docking studies simulated the prenyl transfer reaction of FoPT1. E186 was involved in the formation of prenyl carbonium ion. E98, F89, F182, Y197 and E246 positioned apigenin for catalysis.

Prenylated flavonoids are an important class of phenolics, which combine a flavonoid skeleton with a lipophilic prenyl side chain. Prenylation has been detected on most flavonoids, such as chalcones, flavanones, flavones, flavonols and isoflavones^1^. While flavonoids are usually present at a high level in nature^2^, the levels of prenylated flavonoids are much lower. To date, prenylated flavonoids have been identified in limited plant families. *Leguminosae* and *Moraceae* are the most frequently investigated families and many novel prenylated flavonoids have been explored^3^. Approximately one thousand prenylated flavonoids have been identified from plants. Prenylated flavonones comprise the most common sub-class of prenylated flavonoids and prenylated flavanols are the rarest. *C*-prenylation on flavonoids is much more common than *O*-prenylation.

Prenylated flavonoids are generally more bioactive than their non-prenylated precursors. The mechanism of action is that prenylation increases the lipophilicity of flavonoids, which results in a higher affinity to biological membranes and a better interaction with target proteins^4^. *In planta,* prenylated flavonoids are considered as phytoalexins^5^, compounds that play key roles in physiological behaviors when defending against pathogenic microorganisms. As a class of bioactive compounds, prenylated flavonoids possess a wide variety of bioactivities, such as estrogenic activity, immunomodulatory activity, anticancer activity and antioxidant activity^6^,^7^. Most importantly, this type of chemicals are generally recognized as safe to human body.

As the low levels of prenylated flavonoids in nature limit their application in medicines and nutraceuticals, it may be useful to find alternative synthesis approaches. Chemical synthesis is complicated, inefficient and time-consuming. Harsh synthesis conditions and occurrence of many byproducts make chemical synthesis difficult to be applied in large scale. As an environmentally friendly and efficient synthesis technique^8^,^9^, biocatalysis develops very fast in recent years. The key to synthesizing prenylated flavonoids is to find an efficient and stable flavonoid prenyltransferase. Plant-derived flavonoid prenyltransferases are strictly specific to flavonoid substrates, but are usually membrane-bound proteins and hence insoluble in water^10^. Microbe-derived flavonoid prenyltransferases have poor regiospecificity to flavonoid substates, but their water solubilities are much higher and they are easy to be overexpressed in *E. coli*. Therefore, it is of interest to find a water-soluble flavonoid prenyltransferase with strict substrate specificity.

The fungal family *Fusarium* contains several well known pathogenic species that are capable of causing diseases to economically important plants^11^. *Fusarium* also includes endophytic fungus and can produce special bioactive metabolites, like prenylated phenolics^12^,^13^. The presence of prenyltransferases capable of synthesizing prenylated flavonoids is likely, although no prenyltransferases have been reported in the family. In this work, prenyltransferase candidate genes were obtained via searches of NCBI and *Fusarium oxysporum* genome databases (http://genome.jgi.doe.gov/Fusox1/Fusox1.home.html). A prenyltransferase (FoPT1) was cloned from *Fusarium oxysporum*, and its catalytic properties were investigated.

## Experimental section

### Chemicals

The flavonoids substrates (apigenin, dihydrogenistein, genistein, hesperetin, kaempferol, luteolin and naringenin) were purchased from Aladdin (Shanghai, China). HPLC grade acetonitrile was purchased from ANPEL Laboratory Technologies Inc. (Shanghai, China).

The prenyl donor, dimethylallyl diphosphate (DMAPP), was synthesized according to a previously described method^14^. In brief, solvent A was prepared by adding 2.5 ml of concentrated phosphoric acid into 9.4 ml of acetonitrile. Solvent B was prepared by adding 11 ml of triethylamine into 10 ml of acetonitrile. Solvent A were added into solvent B to obtain bis-triethylammonium phosphate (TEAP) in acetonitrile. 3-Methyl-2-buten-1-ol was mixed with trichloroacetonitrile and TEAP at 37 °C for 5 min. The reaction products were purified by C18 semi-preparative column. DMAPP was eluted by 5% methanol in water, concentrated to dryness, and stored in methanol/10 mM NH_4_OH at −20 °C until use.

### Material and Cloning of FoPT1

*Fusarium oxysporum* f. sp. *cubense* Snyder et Hensen (Foc 4) was purchased from Guangdong Microbiology Culture Center (Guangzhou, China). Potato Dextrose Agar medium(OXOID, UK) was used to cultivate the strain, and the mycelia were collected for total RNA extraction. Total RNA was extracted from the mycelia after 7-day culture by using Hipure Fungal RNA Mini Kit (Magen, China) according to the manufacturer’s instructions. First strand cDNA was reversely transcribed by using Primerscript® II 1^st^ strand cDNA synthesis Kit (Takara, Japan).

The coding sequence of FoPT1 was amplified by using N-terminal and C-terminal primers: 5′-ATGACACAAACAAAACACCTTCAAACG-3′ (FoPT1-F) and 5′-TGTACTTTCGGGTTCTCGAAATGCT-3′ (FoPT1-R).

### Codon optimization of FoPT1

The codon-optimized sequence of FoPT1 was generated by using the Jcat tool (http://www.jcat.de/). The open reading frame was optimized by using the codon bias of *Saccharomyces cerevisiae* and *E. coli*.

### Overexpression and purification of His_6_-FoPT1

The amplified coding region of FoPT1 was cloned into the expression vector pEASY^®^-E2 (TransGen Biotech, China) following the manufacturer’s protocol to give pEASY-FoPT1, with a His_6_ tag at the N-terminus. The recombinant plasmid was transformed into *E. coli* Transetta (DE3) competent cells (TransGen Biotech, China).

*E. coli* Transetta (DE3) strain harboring pEASY-FoPT1 was incubated in 1 L of *Luria-Bertani* medium containing 100 μg/mL ampicillin at 37 °C with a shaking speed of 200 rpm. When the OD_600_ value reached 0.4–0.6, IPTG was added to a final concentration of 0.8 mM to induce the expression of proteins. After 10 h of induction (18 °C, 200 rpm), the cells were harvested by centrifugation (5000 rpm, 10 min, 4 °C).

The cells were re-suspended in lysis buffer (50 mM Tris-HCl, pH 7.5, containing 100 mM NaCl, 10% glycerol and 1 mM PMSF) and then sonicated (40% power, on:5 s, off:10 s, for 30 min) in ice by using an ultrasonic cell disruptor (VCX750, SONICS, USA). The lysate was clarified by centrifugation (9000*g*, 30 min, 4 °C) and filtered through a 0.45 μm membrane. Ni-NTA affinity chromatography (Ni-NTA agarose, QIAGEN, CA, USA) was used to purify the recombinant proteins upon loading the resulting supernatant. The impurities were eluted with wash buffer 2 (50 mM Tris-HCl, pH 7.5, containing 100 mM NaCl and 20 mM imidazole), wash buffer 3 (50 mM Tris-HCl, pH 7.5, containing 100 mM NaCl and 40 mM imidazole), and wash buffer 4 (50 mM Tris-HCl, pH 7.5, containing 100 mM NaCl and 80 mM imidazole). The target proteins were eluted with wash buffer 5 (50 mM Tris-HCl, pH 7.5, containing 100 mM NaCl and 500 mM imidazole). A 10 kDa Amicon Ultra-15 centrifuge filter (Millipore, USA) was used for desalination and a buffer (50 mM Tris-HCl, pH 7.5, containing 10% glycerol and 10 mM DTT) was used to replace the desalted buffer. The purified FoPT1 was stored at −80 °C until use.

The content of proteins was measured by using Bio-Rad Protein Assay Kit (Bio-Rad, USA).

### Assay of FoPT1 activity

Enzyme assays were performed in reaction system vessels (200 μl) containing 50 mM Tris-HCl (pH 7.5), 10 mM divalent cations, 500 μM flavonoid substrates, 1 mM DMAPP and 180 μg of recombinant FoPT1. After incubation for 4 h at 37 °C, the products were extracted by ethyl acetate (3 × 200 μl). The organic layer was evaporated by Concentrator Plus (Eppendorf, Hamburg, Germany) until dryness, and the product was dissolved in 200 μl of methanol for HPLC analysis. For quantification, three repeated assays were routinely carried out.

To test the effects of divalent cations on catalytic activity of FoPT1, CaCl_2_, MgCl_2_, MnCl_2_, NiCl_2_, FeCl_2_, CoCl_2_, and CuCl_2_ were used individually. To confirm the optimal pH, various Tris-HCl buffers (pH 6.6, 7.0, 7.4, 7.8, 8.0, 8.2) were tested. The time courses were also determined.

### Preparation and identification of the reaction products

To prepare the enzymatic products for structure elucidation, the reaction volume was scaled up to 10 mL, containing 50 mM Tris-HCl (pH 7.5), 10 mM CaCl_2_, 500 μM flavonoid substrates (apigenin, dihydrogenistein, genistein, hesperitin, kaempferol, luteolin and naringenin), 1 mM DMAPP and 9 mg of recombinant FoPT1. The reaction vessel was incubated for 4 h at 37 °C. After extraction by ethyl acetate three times, the organic layer was concentrated under vacuum and the products were dissolved in methanol. The enzymatic products were purified by semi-preparative reversed-phase HPLC. The structure of the products were characterized by nuclear magnetic resonnance (NMR) spectroscopy and mass spectrometry (MS) (Bruker AVIII, 500 MHz). Purified products were vacuum evaporated to dryness, dissolved in CD_3_OD.

### HPLC and HPLC-MS/MS analyses of reaction products

The analyses of reaction products and the determinations of the conversion rates were performed on an Agilent 1260 Infinity HPLC system (Agilent Technologies, Germany). The analyses were performed on an Agilent ZORBAX SB-C18 column (3.0 × 100 mm, 1.8 μm, 600 bar). The flow rate was 0.5 ml/min, the column temperature was set at 40 °C, and the injection volume was 10 μl. 280 nm was the monitoring wavelength. The mobile phases comprised solvent A (ultrapure water) and B (acetonitrile). The elution program was as follows: 0–3 min, 10–35% B; 3–20 min, 35–70% B; 20–21 min, 70–100% B; 21–24 min, 100% B.

HPLC-MS/MS was analysed on a maXis LC-ESI-QTOF-MS system (Bruker, Germany) equipped with an Anpel C18 column (4.6 × 150 mm, 5 μm)^15^,^16^. The elution program was the same to HPLC analysis. Ionization of the analytes was achieved by using electron spray ionization interface in positive mode. The collision voltage was 10 eV. Mass scan was set in the range of *m*/*z* 50–1000. The daughter ions were monitored at a collision voltage of 28–42 eV.

### Apparent *K*_*m*_ value assays

To determine the apparent *K*_*m*_ values, the reaction mixture was prepared as above with various concentrations of flavonoids (50–500 μM) and a fixed concentration of DMAPP (1 mM). All experiments were performed in triplicate, and the enzyme activity was evaluated by the formation of prenylflavonoids (nmol) per mg of recombinant protein per minute. Apparent *Km, Kcat* and *Kcat*/*Km* values were calculated from Lineweaver-Burk plots by using Hyper 32 software (http://homepage.ntlworld.com/john.easterby/hyper32.html)^17^.

### Homology modeling and docking of FoPT1

The steric structure of FoPT1 was obtained by homology modeling with the X-ray structure of protein FgaPT2 (SMTL id: 3i4x.1) as a template. Both sequences were aligned by Multalin^18^, and the result was saved as fasta format. The steric structure was generated by Modeller. The best-scored model was visually inspected in order to ascertain sensible structure motifs. Autodocktools was used to dock FoPT1, DMAPP, apigenin and Ca^2+ ^19. The molecular dynamics simulations were run with Ambertools 15.0. Amber coordinate, topology files were obtained by tleap and TIP3PBOX water model. The resulting system was minimized and heated from 0 K to 300 K in 1 ns, equilibrated at 300 K and 1.01 × 10^5^ Pa for 1ns. Finally, regular molecular dynamics simulation for 12 ns was conducted.

## Results and Discussion

### Characterization and cloning of *FoPT1* gene

**Figure 1:**
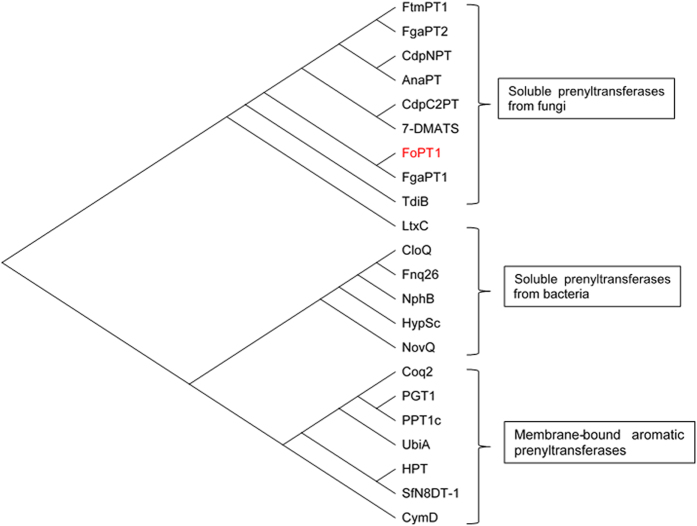
Phylogenetic tree based on amino acid sequences of various prenyltransferases. Amino acid sequences were analyzed using the MEGA 6.0 program. Accession number: FtmPT1, EDP49185; FgaPT2, EEQ33236; CdpNPT, EF433418; AnaPT, EAW16181; CdpC2PT, KF220294; 7-DMATS, Q4WYG3.2; FgaPT1, KMK61245; TdiB, ABU51603; LtxC, AAT12285; CloQ, AAN65239; Fnq26, CAL34104; NphB, BAE00106; HypSc, ACL78791; NovQ, AF170880; Coq2, P32378; PGT1, BAB84122; PPT1c, BAE96574; UbiA, BAB38446; HPT, NP849984; SfN8DT-1, BAG12671; CymD, SARE4565.

A 1,284 bp open reading frame encoding 427 amino acids was cloned from *F. oxysporum*. The open reading frame is shown in Fig. 1S. The calculated molecular mass was 48.5 kDa with a pI of 5.67. The phylogenetic analysis was conducted by neighbor-joining method based on the amino acid sequences of known prenyltransferases. It showed that FoPT1 belonged to the group of water-soluble prenyltransferases from fungi (Fig. 1). However, FoPT1 showed low sequence similarity to other soluble prenyltransferases from fungi. The identities of FoPT1 gene sequence were only 30.5% when comparing with 7-DMATS^20^, 25.1% with AnaPT^21^ and 23% with FgaPT1^22^, respectively.

### Codon optimization of FoPT1

**Figure 2:**
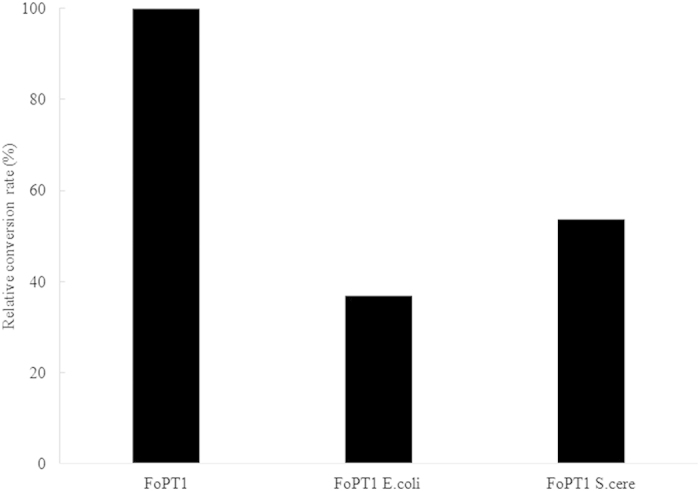
The catalytic efficiency of native and codon-optimized FoPT1. FoPT1, the native gene cloned from *F. oxysporum*; FoPT1 *E.coli*, the gene was optimized by the codon bias of *E. coli*; FoPT1 S.cere, the gene was optimized by the codon bias of *S. cerevisiae*.

As FoPT1 could be actively expressed in *E. coli*, the open reading frame was optimized following the codon usage bias of *E. coli* (FoPT1 *E.coli*). Furthermore, another optimized codon, FoPT1 S.cere was synthesized following the codon usage bias of *S. cerevisiae*. The three genes were cloned into pEASY-E2 plasmid and transformed to *E. coli* Transetta (DE3). Through IPTG induced overexpression, three recombinant proteins were extracted and purified. Their catalytic activities of transferring prenyl to apigenin were measured. As shown in Fig. 2, the native FoPT1 showed the highest activity (100%), followed by FoPT1 S.cere (53.7%) and FoPT1 *E.coli* (36.9%). Therefore, the codons of native FoPT1 gene were selected for further expression and analyses. Though *E. coli* was the expression strain, FoPT1 *E.coli* showed the lowest catalytic activity than native FoPT1 and FoPT1 S.cere. Such phenomenon was also observed for PT1L/PT2, two aromatic prenyltransferases from hop trichome^23^. When expression in *S. cerevisiae*, the catalytic activity was in an increasing order with yeast-optimized PT1L/PT2 <native PT1L/PT2 <*Arabidopsis*-optimized PT1L/PT2. These investigations indicated that codon optimization is applicable for enzyme activity improvement.

### Overexpression and purificationof FoPT1

The coding sequence of native FoPT1 was cloned into the vector pEASY^®^-E2, and the fusion plasmid pEASY-E2-FoPT1 was transformed into *E. coli* Transetta (DE3) competent cells. The water soluble proteins (FoPT1) were obtained from transformants of *E. coli* harboring the expression construct. They were purified with Ni-NTA agarose to give a homogeneous FoPT1, as judged by SDS-PAGE graph (Fig. 2S). The observed molecular mass was about 50 kDa. The protein yield was determined to be 15.0 mg/L culture medium.

### Functional characterization of recombinant FoPT1

**Figure 3:**
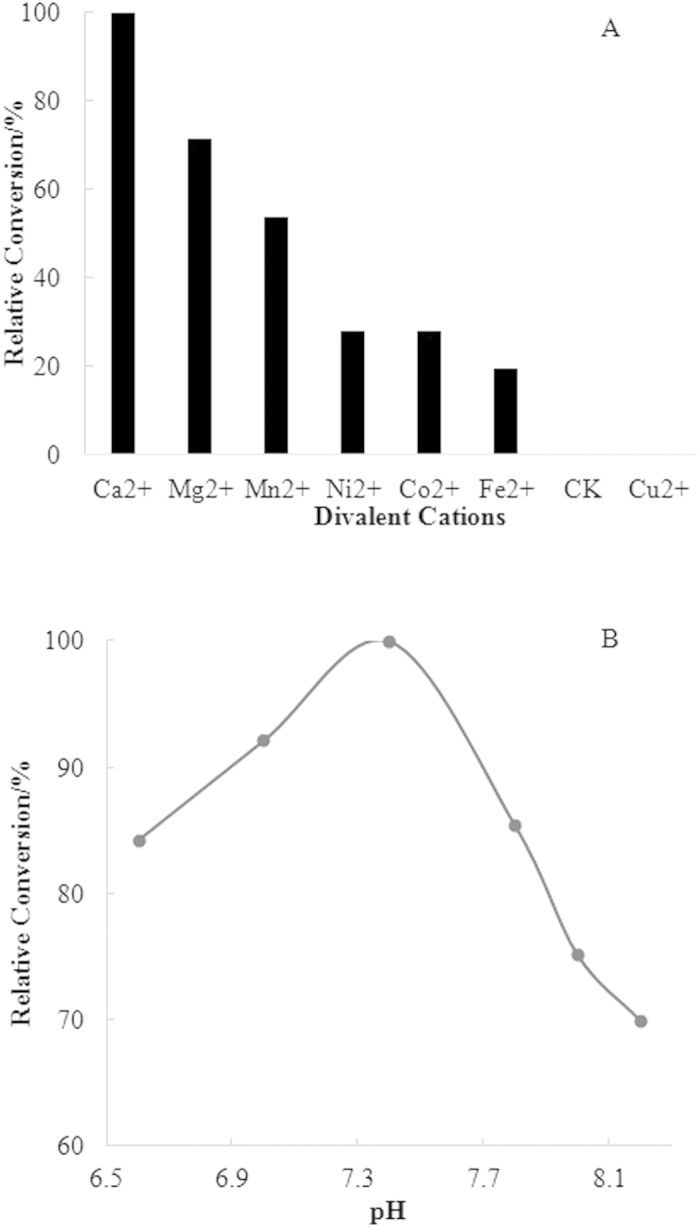
Effects of metal ions and pH value on the catalytic activity of FoPT1. Data were represented as the means of three replicated measurements.

To determine the ion dependency of FoPT1, incubation with different metal ions, including Ca^2+^, Mg^2+^, Ni^2+^, Mn^2+^, Fe^2+^, Co^2+^ and Cu^2+^ at 10 mM, were carried out in the presence of apigenin (**1**) and DMAPP. Incubation without adding metal ions was used as a control. As shown in Fig. 3, the results indicated that the enzyme reaction of FoPT1 was dependent of divalent metal ions. However, different ions had various contributions to the catalytic activity. Ca^2+^ was the most desirable cation for activity, followed by Mg^2+^, Mn^2+^, Ni^2+^, Co^2+^ and Fe^2+^. Addition of Cu^2+^ or no cation addition led to no catalytic activity. Usually, fungal-derived aromatic prenyltransferases are Mg^2+^-dependent (e.g. NphB) or active without divalent cations (e.g. CloQ)^24^.

The effect of pH value on the catalytic activity of FoPT1 was evaluated. A significant change in catalytic activity of FoPT1 was detected in the pH range of 6.6–8.2 (Fig. 3). pH 7.4 was found to be the optimal value to achieve the highest catalytic activity.

**Figure 4:**
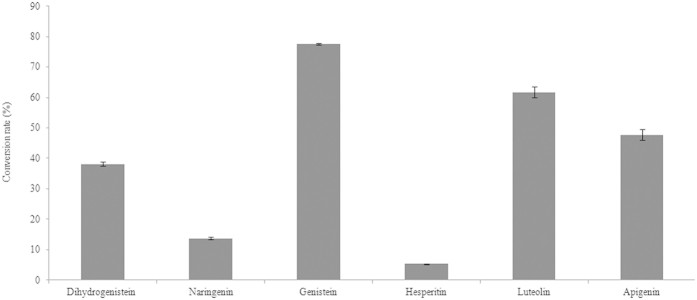
Conversion rate of six flavonoid substrates catalysed by FoPT1. The applied flavonoid concentration was 100 μM.

The time course of kinetics for FoPT1 was determined and the results are shown in Fig. 4S. It showed that the prenylated flavonoids were generated rapidly. Their production reached a steady state when a certain time was applied. For genistein and apigenin, incubation for 360 min would lead to a decrease of prenylated products due to degradation.

The flavonoid substrate specificity of FoPT1 was measured. Apigenin (**1**), kaempferol (**2**), luteolin (**3**), naringenin (**4**), genistein (**5**), dihydrogenistein (**6**), hesperetin (**7**), quercetin (**8**), isoliquiritigenin (**9**), resveratrol (**10**), catechin (**11**), epicatechin (**12**), flavone (**13**), 3-hydroxyflavone (**14**), 5-hydroxyflavone (**15**), 6-hydroxyflavone (**16**), 7-hydroxyflavone (**17**), 5,6,7-trihydroxyflavone (**18**), and dihydrochalcone (**19**) were used to detect the enzymatic activity as flavonoid substrates (Fig. 3S). By HPLC analysis of the reaction products, the product peaks were observed when apigenin (**1**), kaempferol (**2**), luteolin (**3**), naringenin (**4**), genistein (**5**), dihydrogenistein (**6**) and hesperetin (**7**) were used. And the other 12 flavonoid substrates had no products (data not shown).

### Kinetics of FoPT1

Michaelis-Menten kinetics parameters for genistein, luteolin, apigenin, dihydrogenistein, naringenin and hesperetin are listed in Table 1. Michaelis-Menten constant (*K*_*m*_) and turnover number (*K*_*cat*_) were determined from a non-linear fit of initial velocity versus substrate concentration^25^. The Michaelis-Menten constant was not determined for kaempferol due to the low conversion rate. The term *K*_*cat*_/*K*_*m*_ is used as a specificity constant to compare the relative reaction rates of different substrates. *K*_*m*_ is used as an indicator of substrate specificity when [S]/*K*_*m*_ ≪ 1, *K*_*cat*_ is the more relevant indicator when [S]/*K*_*m*_ increases above the value^26^. The order of *K*_*m*_, *K*_*cat*_ and *K*_*cat*_/*K*_*m*_ for six flavonoid substrates were inconsistent. Genistein was regarded as the most active substrate of FoPT1 by *K*_*cat*_ (6.92 × 10^−3^ s^−1^) and *K*_*cat*_/*K*_*m*_ (250.48 s^−1^M^−1^). Hesperetin was the substrate having the lowest affinity to FoPT1 according to the *K*_*cat*_ (5.39 × 10^−4^ s^−1^) and *K*_*cat*_/*K*_*m*_ (22.13 s^−1^M^−1^).

The conversion rates were calculated as shown in Fig. 4. No prenylated products were observed when the kaempferol concentration was less than 500 μM, and the conversion rate was less than 1% when 500 μM kaempferol was used. When 100 μM flavonoid was applied, the conversion rate was in a decreasing order with genistein >luteolin >apigenin >dihydrogenistein >naringenin >hesperetin >kaempferol.

### The structure requirements of flavonoid as substrate of FoPT1

By comparing the structure differences of preferred flavonoid substrates, it could be concluded that 5,7-dihydroxyl and 4-carbonyl groups of flavonoid were required for the prenylation reaction. It has been reported that 5,7-dihydroxy and 3′,4′-dihydroxy are beneficial for the prenylation of flavanones by SfFPT^27^. The beneficial effect of 3′,4′-dihydroxy was not observed in this work, as quercetin could not act as substrate while kaempferol could. 3-Hydroxy significantly impaired the conversion rate as quercetin could not be prenylated by FoPT1 and the conversion of kaempferol could be detected. By comparing the conversion rate of genistein/dihydrogenistein and naringenin/apigenin, it was obvious that 2,3-alkenyl in flavonoid skeleton was beneficial for catalysis.

As it was possible that the regioselectivity could be shifted when C-6 of flavonoid was substituted by hydroxy, 5,6,7-trihydroxyflavone was tested to see if the regioselectivity of FoPT1 could be changed. However, no products were observed. FgaPT2 can catalyse the transfer reaction of a benzyl moiety from benzyl diphosphate to C-5 of tryptophan. When C-5 is blocked by hydroxy, the benzylation is taken place at C-6^28^.

### Identification of the reaction products

**Figure 5:**
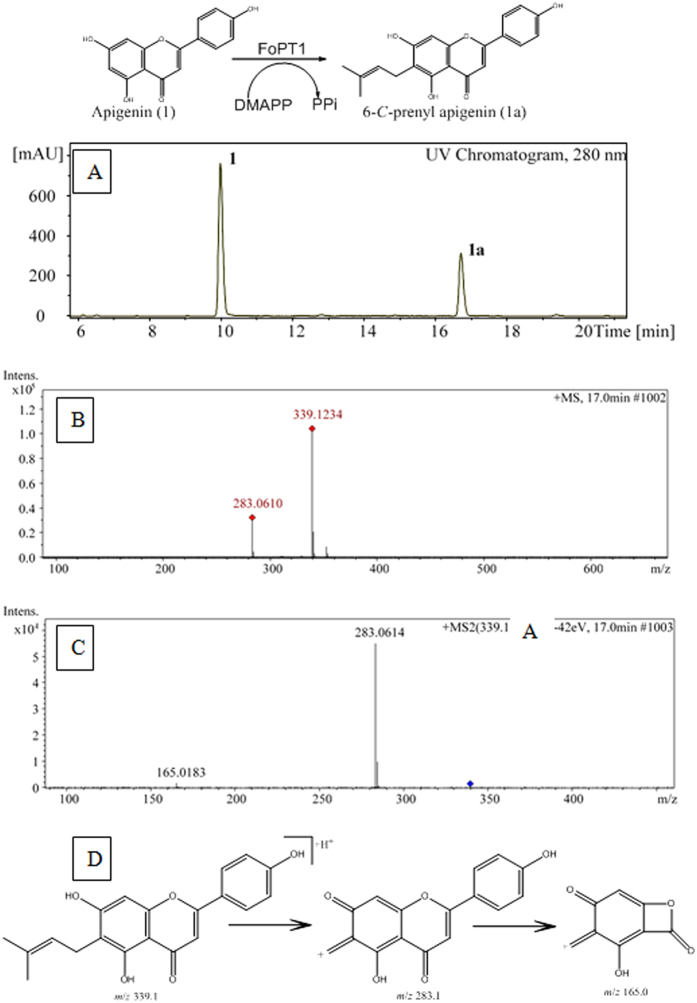
The prenylated product of apigenin analysed by HPLC-MS/MS. (**A**) the chromatogram of the prenylated product recorded at 280 nm; (**B**) the parent ion of 6-*C*-prenyl apigenin at positive mode; (**C**) the daughter ions of 6-*C*-prenyl apigenin at positive mode; (**D**) The putative degradation pathway of 6-*C*-prenyl apigenin.

**Figure 6:**
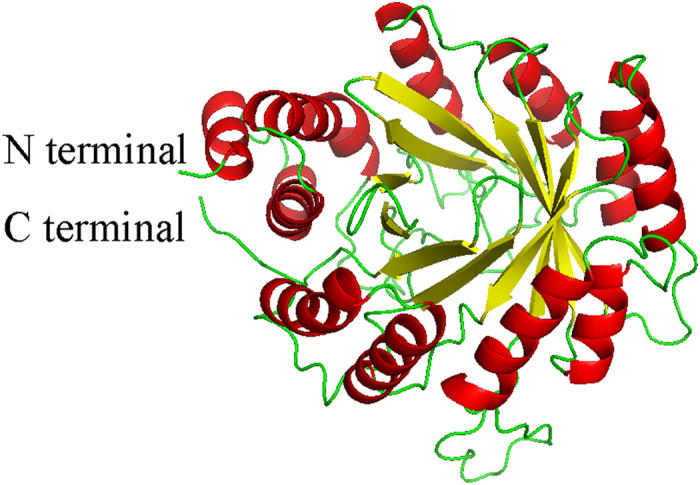
The steric structure of FoPT1. The α-helices were in red, β-strands were in yellow.

**Figure 7:**
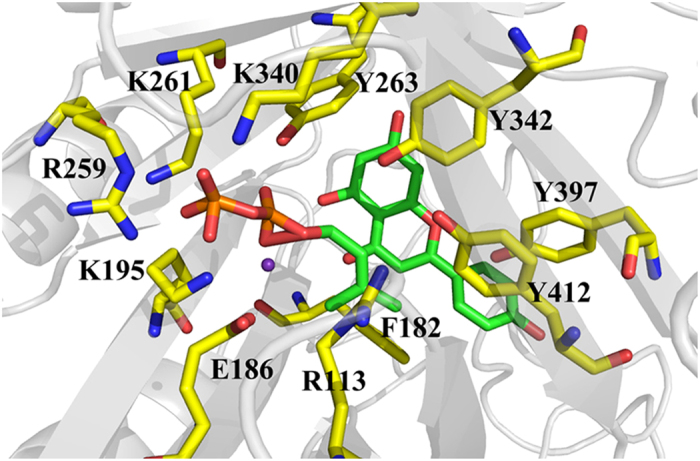
The docking model of FoPT1, apigenin, DMAPP and Ca^2+^.

Only one product was generated when catalyzing 1–7 by FoPT1. **1a**–**7a** were purified by using HPLC in semi-preparative scale and their structures were identified by NMR spectroscopy and mass spectrometry. The 1D and 2D NMR spectra were recorded, and the chemical shifts were assigned as shown in Table 1S. 6-*C*-prenyl apigenin (**1a**) was identified as below. Signals of a prenyl were observed for C-1″/H-1″ (22.0/3.31), C-2″/H-2″ (123.0/5.23), C-3″ (130.6), C-4″/H-4″ (methyl, 17.8/1.78) and C-5″/H-5″ (methyl, 25.8/1.68). HMBC spectra showed a correlation between H-1″ and C-5 (159.7) as well as C-7 (162.5). It indicated that C-1″ of prenyl was linked to C-6 of apigenin. A shift to low field was detected for H-8 when the prenyl was substituted at C-6. HPLC-MS/MS was used to analyse the reaction product. Figure 5 shows the chromatogram at 280 nm and MS/MS spectra of 6-*C*-prenyl apigenin. A small product peak was occurred at 17.0 min, and the [M + H]^+^ of parent ion was determined to be *m*/*z* 339.1234 in positive mode. It was consistent with the molecular weight of prenyl apigenin. The daughter ions were obtained when the collision votage was set to 28–42 eV. The presence of [M + H]^+^ *m*/*z* at 283.0614 suggested the cleavage of prenyl between C-1” and C-2”^29^. [M + H]^+^ *m*/*z* at 165.0183 indicated the further breakage of C ring to form a A^1,3^ fragment ion^30^. This fragment ion confirmed that the prenyl was located at the A ring. The degradation pathway of 6-*C*-prenyl apigenin was hypothesized as shown in Fig. 5D. The other six reaction products were also identified by HPLC-MS/MS as above. Please see Figs 5S–10S.

According to the binding location of prenyl donor, reverse and regular prenyltransferases are defined. A C-C bond between C-1 of the prenyl diphophate and an aromatic nucleus is formed by regular prenyltransferase, while a C-C bond between C-3 of the prenyl diphophate and an aromatic nucleus is formed by reverse prenyltransferase^22^. As shown in the phylogenetic tree (Fig. 1), FgaPT1 had the highest similarity to FoPT1. It has been reported that FgaPT1 catalyzes a reverse prenylation at C-2 of the indole moiety in tryptophan^31^. However, FoPT1 showed a different prenylation type. It was a regular prenyltransferase generating a C-C bond between C-1 of DMAPP and C-6 of flavonoids.

Flavonoid prenyltransferases from other fungal and bacterial sources have been reported before. NovQ from *Streptomyces niveus* is capable of catalyzing the transfer of a dimethylallyl group to both phenylpropanoids and B-ring of flavonoids^32^. Usually two prenylated products are generated at C-3′ or O-4′ in the B-ring when genistein and naringenin served as substrates. NphB from *Streptomyces* sp. Strain Cl190 can transfer a geranyl group to C-6, O-7 and C-8 in A-ring of flavonoids^33^. The geranyl location is dependent on the structure of flavonoid. Seven fungal prenyltransferases, including 7-DMATS, AnaPT, CdpC3PT, CdpNPT, SirD, FtmPT1 and FgaPT2, have been tested on thirty flavonoids. 7-DMATS from *Aspergillus fumigatus* shows a more flexible substrate specificity towards flavonoids. It can prenylate the flavonoid at C-6, C-2′ and C-5′^34^.

### Regiospecificity of FoPT1

Through structure identification of **1a**–**7a**, all these products were prenylated at C-6. No products prenylated at other locations were detected. This indicated that FoPT1 was a highly regiospecific prenyltransferase. This was different from other prenyltransferases cloned from fungi as they usually generates multiple prenylated flavonoids.

The dimethylallyl tryptophan synthase (DMATS) family is involved in catalysing the reaction to transfer dimethylallyl moiety from DMAPP to various positions of the indole ring of tryptophan^35^. Members of this family have high flexibility towards aromatic substrates, and flavonoids can be acted as prenyl acceptors. However, more than one prenylated products will be generated via catalysis with DMATS. When eriodictyol is prenylated by a 7-DMATS, five products, including 6-*C*-prenyl eriodictyol (conversion rate 22.8%), 5′-*C*-prenyl eriodictyol (conversion rate 31.1%) and three other unidentified prenylated products, were observed^36^. Such catalysis behavior makes purification of the final prenylated flavonoids difficult. Therefore, it is of interest to find a highly regiospecific prenyltransferase, and FoPT1 is the one mentioned in this work.

### Homology modeling of FoPT1

To get insights into the active sites of FoPT1, the 3D structure of FoPT1 protein was obtained by homology modeling. AnaPT^37^, FgaPT2^26^, FtmPT1^38^ and CdpNPT^39^ were the applicable templates when searching in SWISS-MODEL. FgaPT2 was chosen to be the most suitable template. The 3D structure of FoPT1 was consisted of a central barrel of ten *β*-sheets that were surrounded with a ring made by eleven *a*-helices (Fig. 6), which was called PT barrel. FoPT1 comprised of five structurally similar repeating units, e.g. ααββ units. Two *a*-helices packed tightly against two *β*-strands in each repeat, and they formed a hydrophobic core. An extra N-terminal *a*-helix was observed for FoPT1, and it was similar to FgaPT2. Through alignment with FgaPT2, the active site in FoPT1 binding prenyl donor included R113, E186, K195, Y197, R259, K261, Y263, V325, K340, Y342, Y391, Q403 and Y412. Mutations of four residues, V325M, K340Q, Q403R and S408Y, replaced the diphophate binding site of FoPT1 with that of FgaPT2^31^. The active site binding prenyl receptor of FoPT1 contained F89, M90, E98, A115, F182, Y197, I199, F246, V325, Y397 and Y412. Mutations of seven residues, F89I, M90L, A115T, F182K, I199Y, F246R and V325M, replaced the diphophate binding site of FoPT1 with that of FgaPT2^31^.

### Docking with DMAPP and apigenin

The docking model between FoPT1, DMAPP, apigenin and Ca^2+^ was constructed. The model with the lowest binding energy was selected, which was calculated to be −7.46 kcal/mol. Molecular dynamics calculation was conducted to evaluate the precision of the model. The docking model after balancing for 8–12 ns was used. As shown in Fig. 7, Ca^2+^ was coordinated by E186 and DMAPP, which was involved in the removal of diphosphate in DMAPP and formation of carbonium ion. A strong hydrogen bond was formed between apigenin and E98. Other amino acid residues, such as F89, F182, Y197 and F246, positioned the apigenin through hydrophobic forces. Apigenin and DMAPP located closely to each other in the docking model, making the electrophilic attack of dimethylallyl carbonium ion to C-6 of apigenin possible.

Mg^2+^ is usually required for the catalysis of membrane-embedded prenyltransferase from the UbiA family^40^. Family members typically contain eight or nine transmembrane helices and two characteristic conserved motifs with the consensus sequences NDXXDXXXD and DXXXD. FoPT1 is an ABBA type aromatic prenyltransferase, and this family has no Asp-rich motifs for substrate binding and has a poor substrate specificity^41^. Mg^2+^-dependent and -independent prenyltransferases are both present in this family. Replacement of the Mg^2+^ cofactor with basic residues yields a similar activation barrier for prenylation to Mg^2+^-dependent prenyltransferase like NphB^41^. In this work, FoPT1 was cofactor-dependent, and Ca^2+^ acted more efficiently than Mg^2+^. The above docking model showed that Ca^2+^ was coordinated by the carboxyl of E186 and the oxygen of pyrophosphate group. Once the planar allylic carbocation was generated, it was stabilized by cation-π interactions with aromatic rings from apigenin and Y342. Moreover, Y197, Y263, Y342, Y391 and Y412 prevented the dimethylallyl carbonium ion from reacting with other nucleophiles.

## Conclusions

FoPT1 was confirmed to be a highly regiospecific prenyltransferase. It could recognize apigenin, naringenin, genistein, dihydrogenistein, kampferol, luteolin and hesperetin as substrate, and only 6-*C*-prenylated flavonoids were generated. Chalcones, flavanols and stilbenes could not act as the substrate. 5,7-Dihydroxy and 4-carbonyl of the flavonoid skeleton were required for the catalysis. 2,3-alkenyl was beneficial to the catalysis whereas 3-hydroxy impaired the reaction.

## Additional Information

**How to cite this article**: Yang, X. *et al*. Regiospecific synthesis of prenylated flavonoids by a prenyltransferase cloned from *Fusarium oxysporum. Sci. Rep.* **6**, 24819; doi: 10.1038/srep24819 (2016).

## Supplementary Material

Supplementary Information

## Figures and Tables

**Table 1:** Kinetic parameters of FoPT1 with flavonoid substrates.

Substrate	*K*_*m*_(μM)	V_max_ (μmol min^−1^mg^−1^)	*k*_*cat*_(s^−1^)	*k*_*cat*_/*K*_*m*_(s^−1^M^−1^)	(*k*_*cat*_/*K*_*m*_)_Rel_ (%)
Dihydrogenistein	59.22	0.28	4.61E-03	77.86	31.08
Naringenin	24.80	0.08	1.32E-03	53.38	21.31
Genistein	27.63	0.42	6.92E-03	250.48	100.00
Hesperetin	24.38	0.03	5.39E-04	22.13	8.84
Luteolin	46.31	0.36	6.01E-03	129.68	51.77
Apigenin	25.21	0.21	3.58E-03	141.87	56.64
Kaempferol	N.D	N.D	N.D	N.D	N.D
