# Supplementary material for: Regiospecific synthesis of prenylated flavonoids by a prenyltransferase cloned from Fusarium oxysporum
Source: Sci Rep. 2016 Apr 21;6:24819. doi: 10.1038/srep24819 (PMC4838938; doi:10.1038/srep24819)
Supplement: Supplementary Information [file srep24819-s1.pdf]

Supplementary information

Regiospecific synthesis of prenylated flavonoids by a prenyltransferase cloned from *Fusarium oxysporum*

Xiaoman Yang[a,#], Jiali Yang[a,#], Yueming Jiang[a], Hongshun Yang[b], Ze Yun[a], Weiliang Rong[a], Bao Yang[a,*]

* Corresponding author

Bao Yang, PhD, Professor

Tel.: +86 20 37083042

E-mail: yangbao@scbg.ac.cn

Supplemented information

Table 1S. NMR assignments of **1a**-**7a** in CD_3OD.

Figure 1S. Coding sequences of native and codon-optimized FoPT1.

Figure 2S. Proteins distribution on SDS-PAGE. Lane 1, standard protein ladder; Lane 2, total soluble proteins; Lane 3, flow-through fraction when loading crude proteins; Lane 4, washing buffer 2-eluted fraction; Lane 5, washing buffer 3-eluted fraction; Lane 6, washing buffer 4-eluted fraction; Lane 7, washing buffer 5-eluted fraction (purified FoPT1-His_6).

Figure 3S. the chemical structure of flavonoids used in this work.

Figure 4S. Effect of time on the production of prenylated flavonoids.

Figure 5S. The prenylated product of kaempferol analysed by HPLC-MS/MS. A, the chromatogram of the prenylated product recorded at 280 nm; B, the parent ion of 6-*C*-prenyl kaempferol at positive mode; C, the daughter ions of 6-*C*-prenyl kaempferol at positive mode.

Figure 6S. The prenylated product of luteolin analysed by HPLC-MS/MS. A, the chromatogram of the prenylated product recorded at 280 nm; B, the parent ion of 6-*C*-prenyl luteolin at positive mode; C, the daughter ions of 6-*C*-prenyl luteolin at positive mode.

Figure 7S. The prenylated product of naringenin analysed by HPLC-MS/MS. A, the chromatogram of the prenylated product recorded at 280 nm; B, the parent ion of 6-*C*-prenyl naringenin at positive mode; C, the daughter ions of 6-*C*-prenyl naringenin

at positive mode.

Figure 8S. The prenylated product of genistein analysed by HPLC-MS/MS. A, the chromatogram of the prenylated product recorded at 280 nm; B, the parent ion of 6-*C*-prenyl genistein at positive mode; C, the daughter ions of 6-*C*-prenyl genistein at positive mode.

Figure 9S. The prenylated product of dihydrogenistein analysed by HPLC-MS/MS. A, the chromatogram of the prenylated product recorded at 280 nm; B, the parent ion of 6-*C*-prenyl dihydrogenistein at positive mode; C, the daughter ions of 6-*C*-prenyl dihydrogenistein at positive mode.

Figure 10S. The prenylated product of hesperitin analysed by HPLC-MS/MS. A, the chromatogram of the prenylated product recorded at 280 nm; B, the parent ion of 6-*C*-prenyl hesperitin at positive mode; C, the daughter ions of 6-*C*-prenyl hesperitin at positive mode.

Table 1S. NMR assignments of **1a**-**7a** in CD_3OD.

	6-*C*-prenyl apigenin, **1a**		6-*C*-prenyl kaempferol, **2a**		6-*C*-prenyl luteolin, **3a**		6-*C*-prenyl naringenin, **4a**	
	C	H	C	H	C	H	C	H
2	164.5		146.8		164.9		79.5	5.35,dd,3.0,13.0Hz
3	103.5	6.58,s	135.6		103.6	6.54	43.3	2.69,dd,3.0,17.5Hz<br>3.30,dd,13.0,17.5Hz
4	182.8		175.9		182.4		196.6	
4a	104.8		103.0		104.4		102.6	
5	159.7		160.4		160.5		164.1	
6	112.0		109.5		109.5		109.3	
7	162.5		163.5		163.6		164.9	
8	93.8	6.49,s	93.8	6.48,s	93.9	6.48	95.4	5.94,s
8a	156.2		156.1		157.9		162.3	
1’	122.9		121.6		122.4		128.6	
2’	128.8	7.88,dd,8.0,2.0Hz	129.5	8.11,dd,2.0,8.0Hz	113.9	7.37,d,2.5Hz	128.8	7.31,dd,8.5,2.0Hz
3’	116.4	6.98,dd,8.0,2.0Hz	115.4	6.90,dd,2.0,8.0Hz	146.4		116.2	6.82, dd,8.5,2.0Hz
4’	161.7		159.1		150.5		158.3	
5’	116.4	6.98,dd,8.0,2.0Hz	115.4	6.90,dd,2.0,8.0Hz	116.6	6.90,d,8.0Hz	116.2	6.82, dd,8.5,2.0Hz
6’	128.7	7.88,dd,8.0,2.0Hz	129.5	8.11,dd,2.0,8.0Hz	120.0	7.38,dd,8.0,2.5Hz	128.8	7.31,dd,8.5,2.0Hz
1’’	22.0	3.31,overlapped	22.2	3.31,overlapped	22.2	3.31,overlapped	21.9	3.21,d,7.5Hz
2’’	123.0	5.23,m	123.2	5.24,m	123.2	5.24,m	123.8	5.19,m
3’’	130.6		130.7		130.7		131.1	
4’’	17.8	1.78,s	17.7	1.79,s	17.8	1.79,s	17.8	1.75,s
5’’	25.8	1.68,s	25.9	1.66,s	25.9	1.66,s	26.4	1.66,s

Table 1S-continued. NMR assignment of **1a**-**7a** in CD_3OD.

	6-*C*-prenyl genistein, **5a**		6-*C*-prenyl dihydrogenistein, **6a**		6-*C*-prenyl hesperitin, **7a**	
	C	H	C	H	C	H
2	153.9	8.04,s	72.9	4.46,dd,11.5,5.0Hz<br>4.50,dd,11.5,5.0Hz	164.9	
3	123.7		52.0	3.86,dd,5.0Hz	103.6	6.54,s
4	183.0		197.8		182.4	
4a	106.6		103.5		104.4	
5	162.6		162.8		160.5	
6	109.1		109.8		109.5	
7	165.8		166.2		163.6	
8	94.0	6.38,s	93.4	5.93,s	93.9	6.48,s
8a	156.1		158.2		157.9	
1’	122.7		128.2		122.1	
2’	131.6	7.37,dd,9.0,2.0Hz	130.9	7.09,dd,2.0,8.5Hz	113.5	7.43,d,2.5Hz
3’	116.4	6.84,dd,9.0,2.0Hz	116.6	6.75,dd,2.0,8.5Hz	147.4	
4’	158.0		158.2		153.4	
5’	116.4	6.84,dd,9.0,2.0Hz	116.6	6.75,dd,2.0,8.5Hz	111.6	6.85,d,8.0Hz
6’	131.6	7.37,dd,9.0,2.0Hz	130.9	7.09,dd,2.0,8.5Hz	119.4	7.44,dd,8.0,2.5Hz
1’’	22.5	3.31,overlapped	22.1	3.20,d,7.0Hz	22.2	3.31,overlapped
2’’	123.5	5.23,m	124.0	5.19,m	123.2	5.24,m
3’’	131.6		131.8		131.2	
4’’	18.1	1.78,s	18.0	1.75,s	17.8	1.79,s
5’’	26.2	1.66,s	26.2	1.65,s	25.9	1.66,s

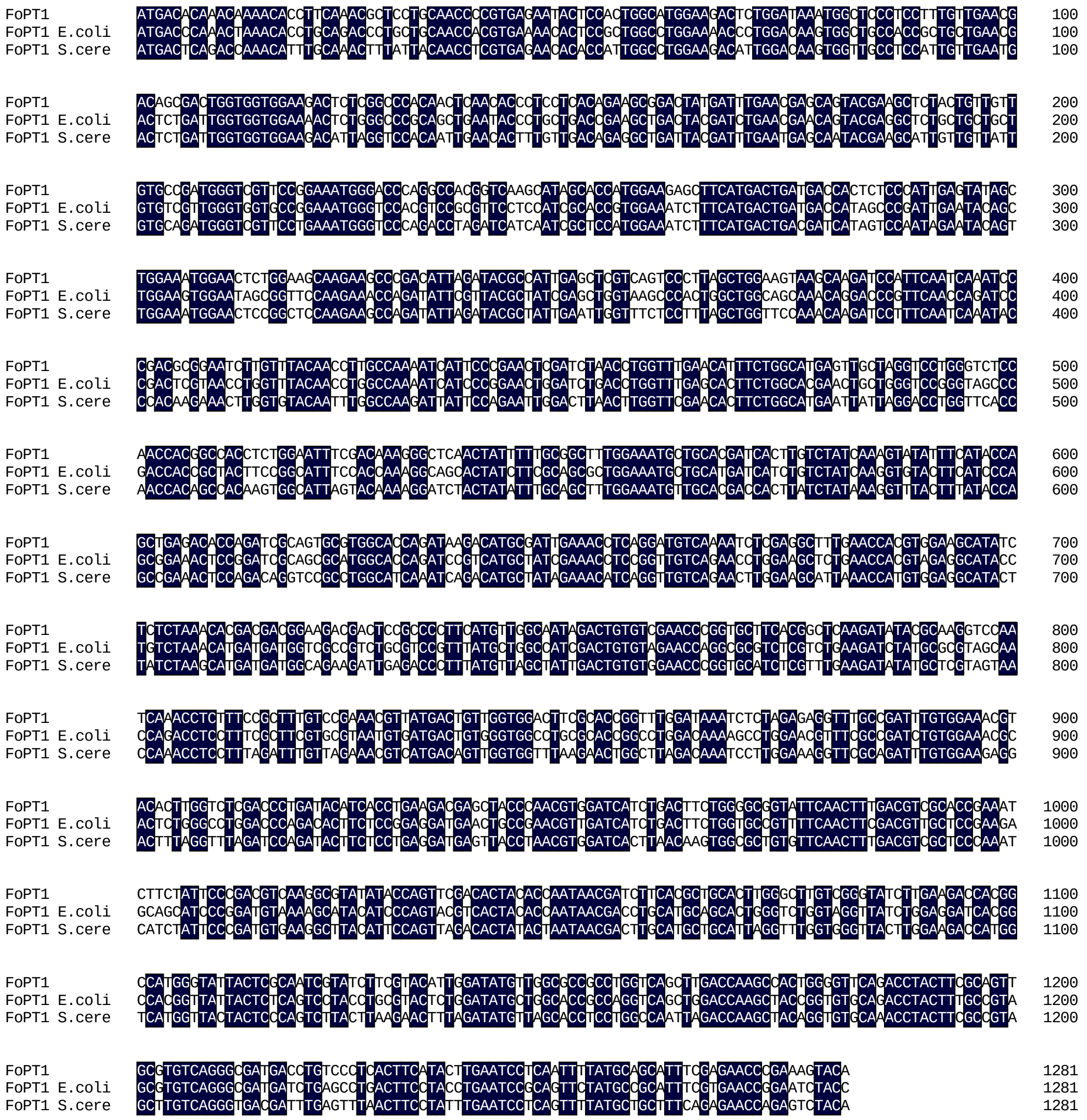

Figure 1S. Nucleotide sequences of native and codon-optimized FoPT1.

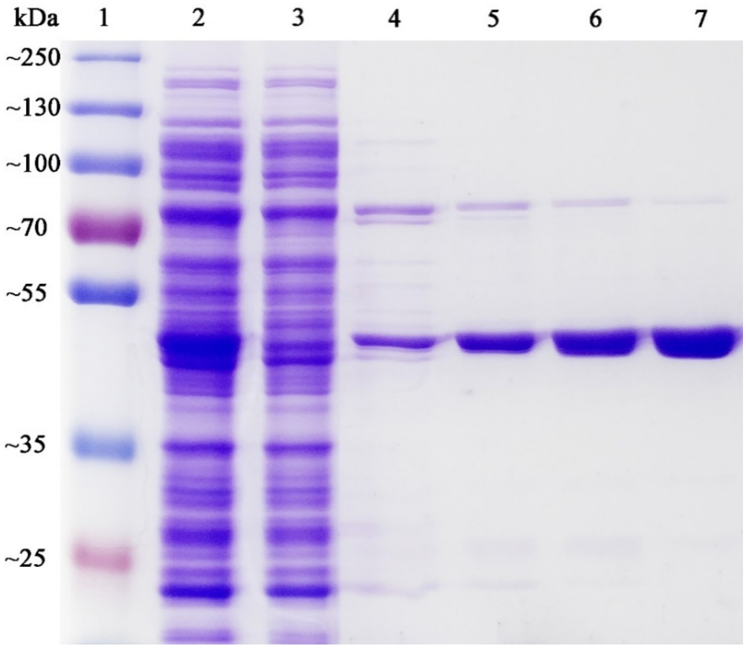

Figure 2S. Proteins distribution on SDS-PAGE. Lane 1, standard protein ladder; Lane 2, total soluble proteins; Lane 3, flow-through fraction when loading crude proteins; Lane 4, washing buffer 2-eluted fraction; Lane 5, washing buffer 3-eluted fraction; Lane 6, washing buffer 4-eluted fraction; Lane 7, washing buffer 5-eluted fraction (purified FoPT1-His_6).

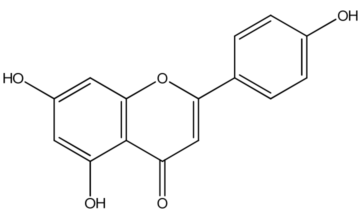

1 apigenin

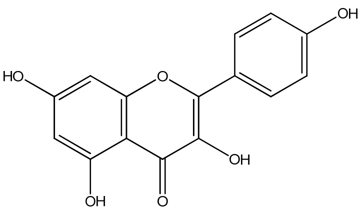

2 kaempferol

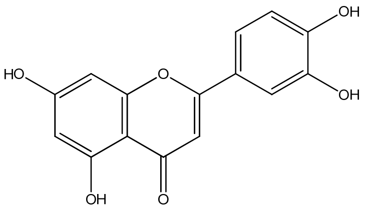

3 luteolin

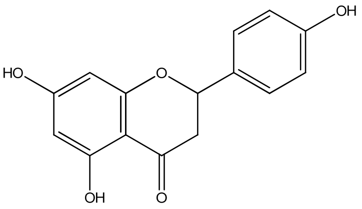

4 naringenin

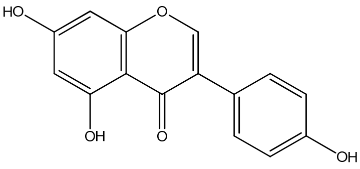

5 genistein

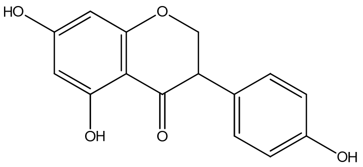

6 dihydrogenistein

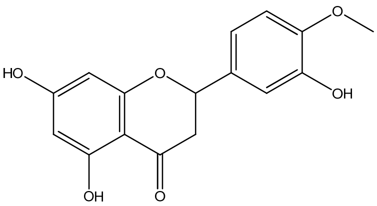

7 hesperetin

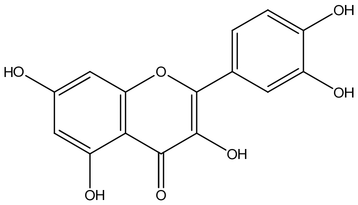

8 quercetin

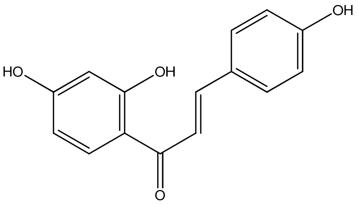

9 isoliquiritigenin

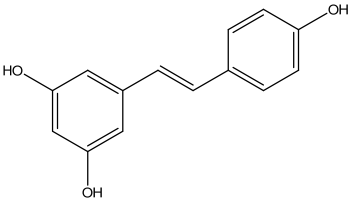

10 resveratrol

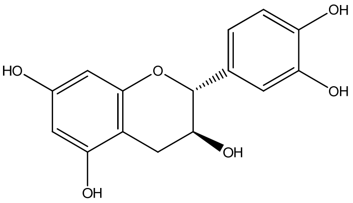

11 (+)-catechin

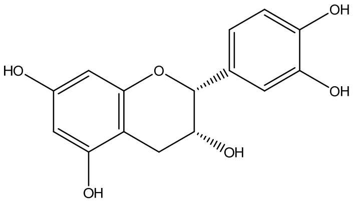

12 (-)-epicatechin

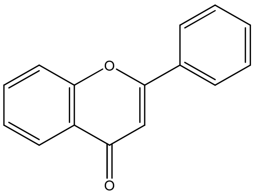

13 flavone

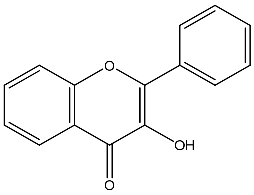

14 3-hydroxyflavone

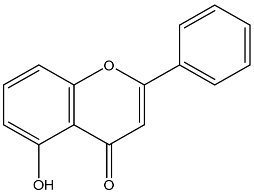

15 5-hydroxyflavone

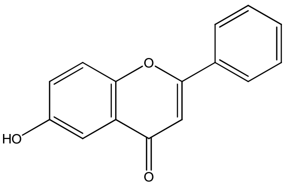

16 6-hydroxyflavone

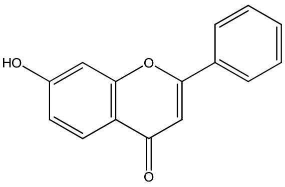

17 7-hydroxyflavone

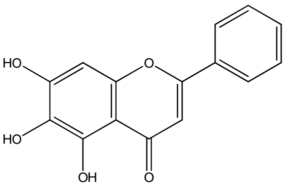

18 5,6,7-trihydroxyflavone

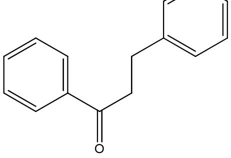

19 dihydrochalcone

Figure 3S. the chemical structure of flavonoids used in this work.

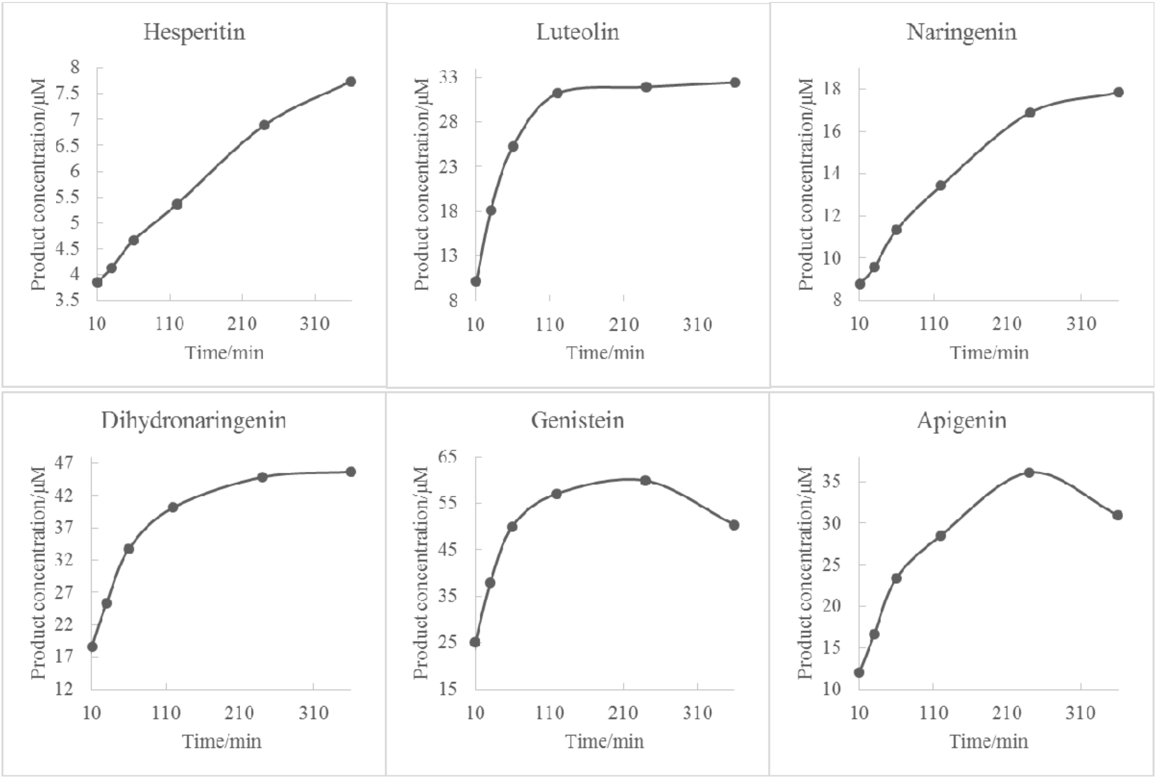

Figure 4S. Effect of time on the production of prenylated flavonoids.

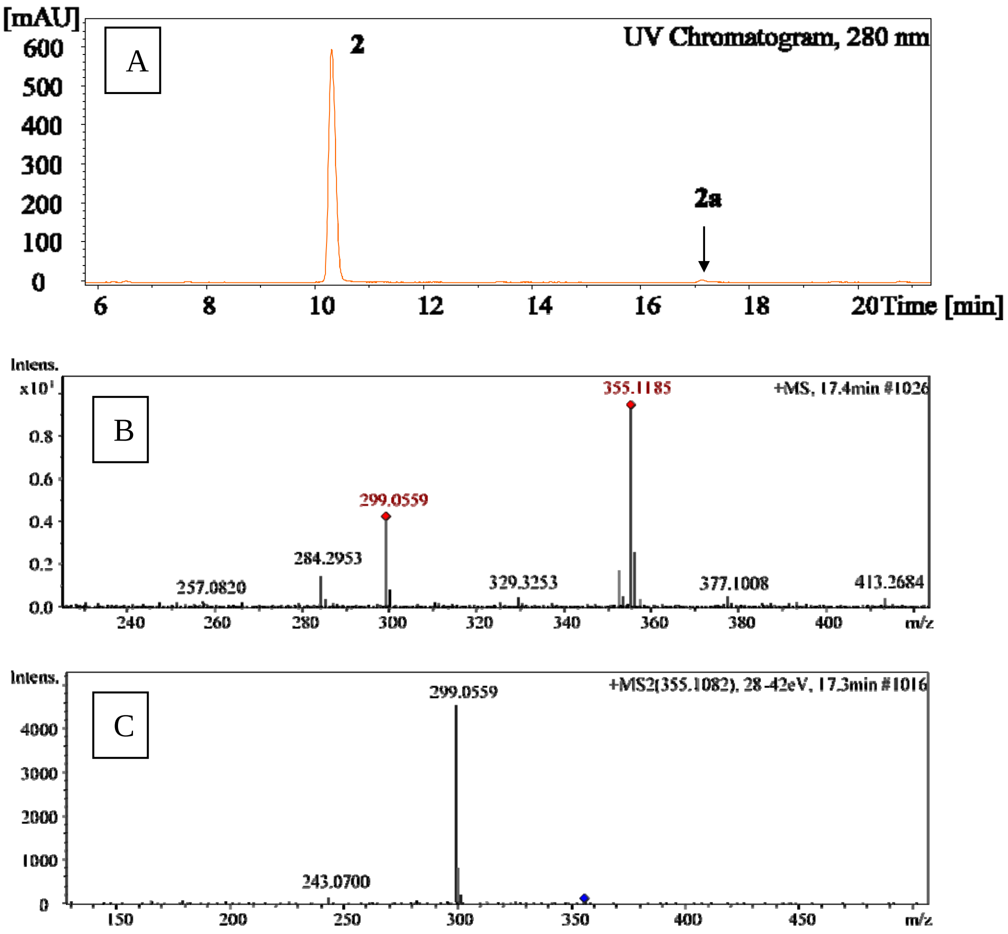

Figure 5S. The prenylated product of kaempferol analysed by HPLC-MS/MS. A, the chromatogram of the prenylated product recorded at 280 nm; B, the parent ion of 6-*C*-prenyl kaempferol at positive mode; C, the daughter ions of 6-*C*-prenyl kaempferol at positive mode.

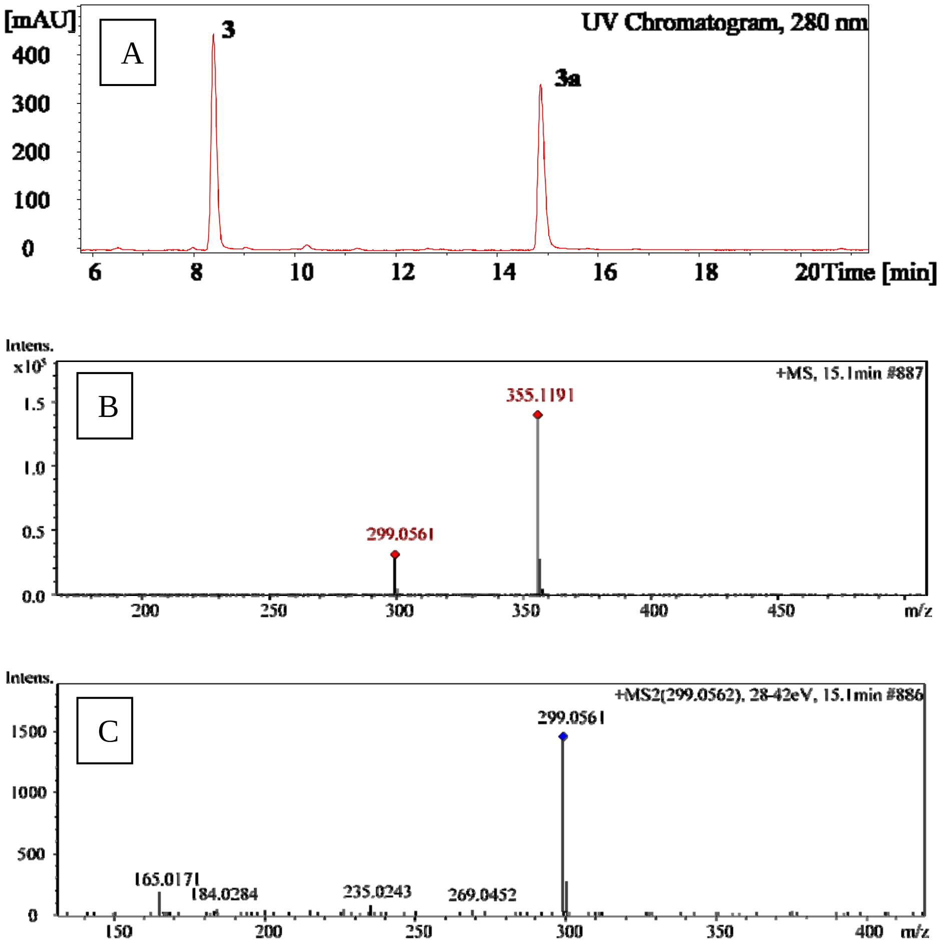

Figure 6S. The prenylated product of luteolin analysed by HPLC-MS/MS. A, the chromatogram of the prenylated product recorded at 280 nm; B, the parent ion of 6-*C*-prenyl luteolin at positive mode; C, the daughter ions of 6-*C*-prenyl luteolin at positive mode.

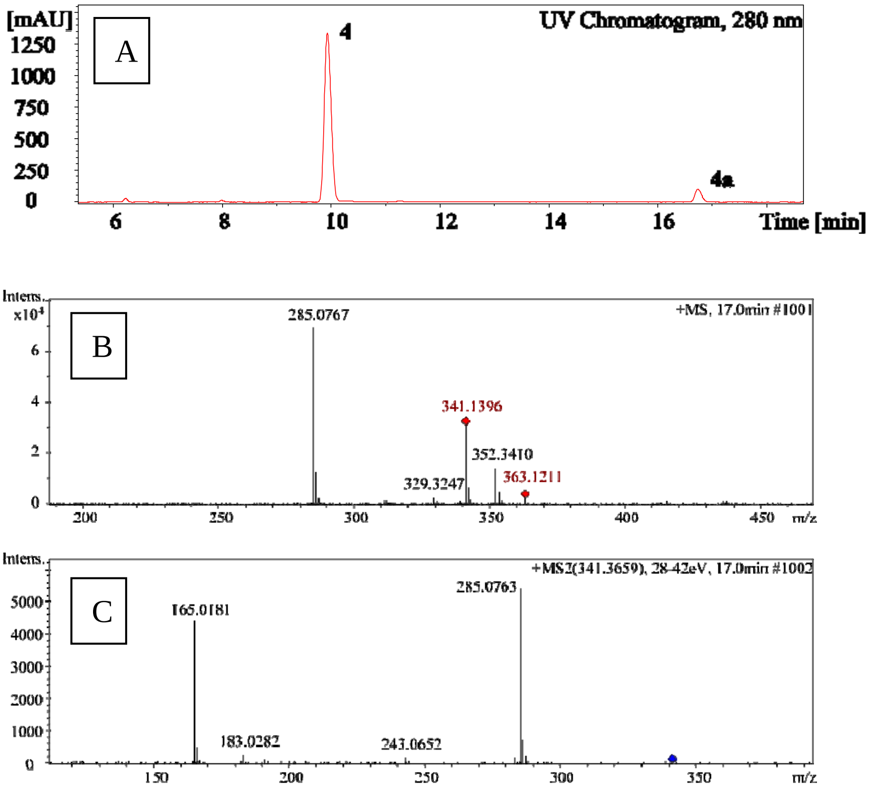

Figure 7S. The prenylated product of naringenin analysed by HPLC-MS/MS. A, the chromatogram of the prenylated product recorded at 280 nm; B, the parent ion of 6-*C*-prenyl naringenin at positive mode; C, the daughter ions of 6-*C*-prenyl naringenin at positive mode.

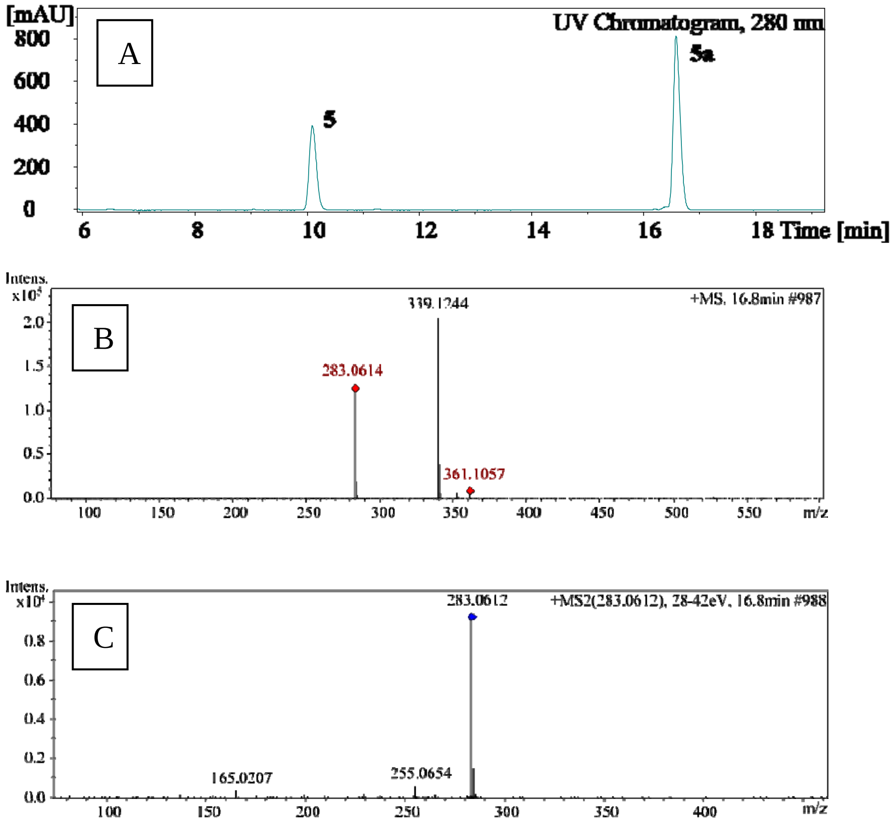

Figure 8S. The prenylated product of genistein analysed by HPLC-MS/MS. A, the chromatogram of the prenylated product recorded at 280 nm; B, the parent ion of 6-*C*-prenyl genistein at positive mode; C, the daughter ions of 6-*C*-prenyl genistein at positive mode.

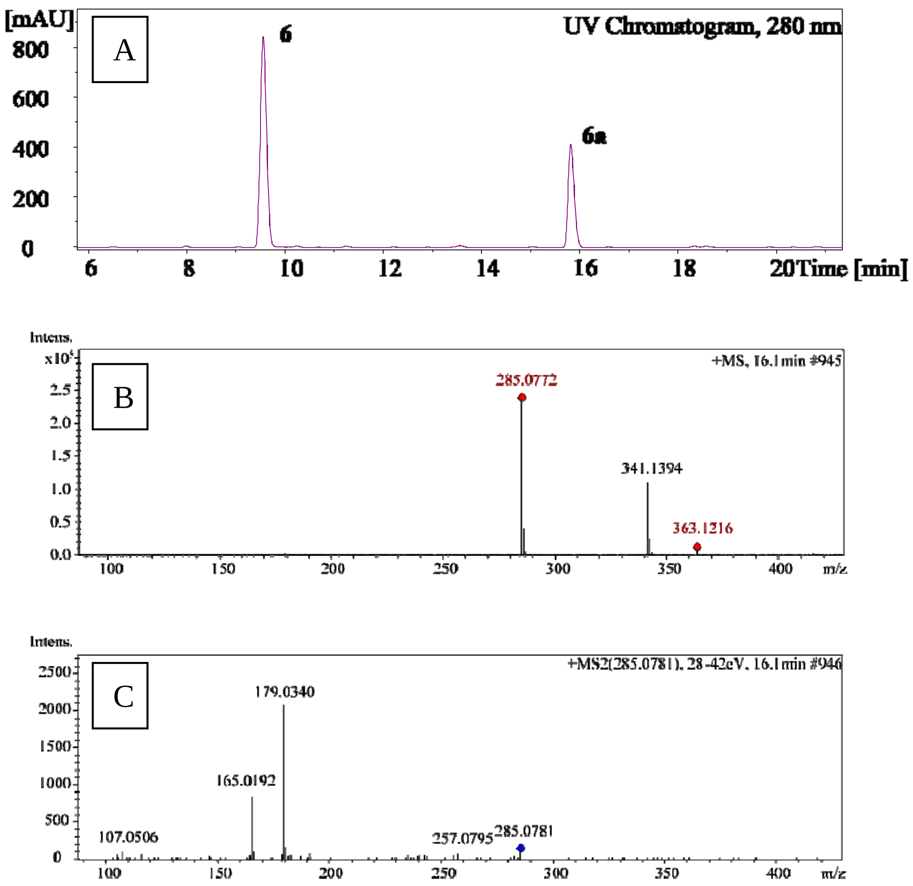

Figure 9S. The prenylated product of dihydrogenistein analysed by HPLC-MS/MS. A, the chromatogram of the prenylated product recorded at 280 nm; B, the parent ion of 6-*C*-prenyl dihydrogenistein at positive mode; C, the daughter ions of 6-*C*-prenyl dihydrogenistein at positive mode.

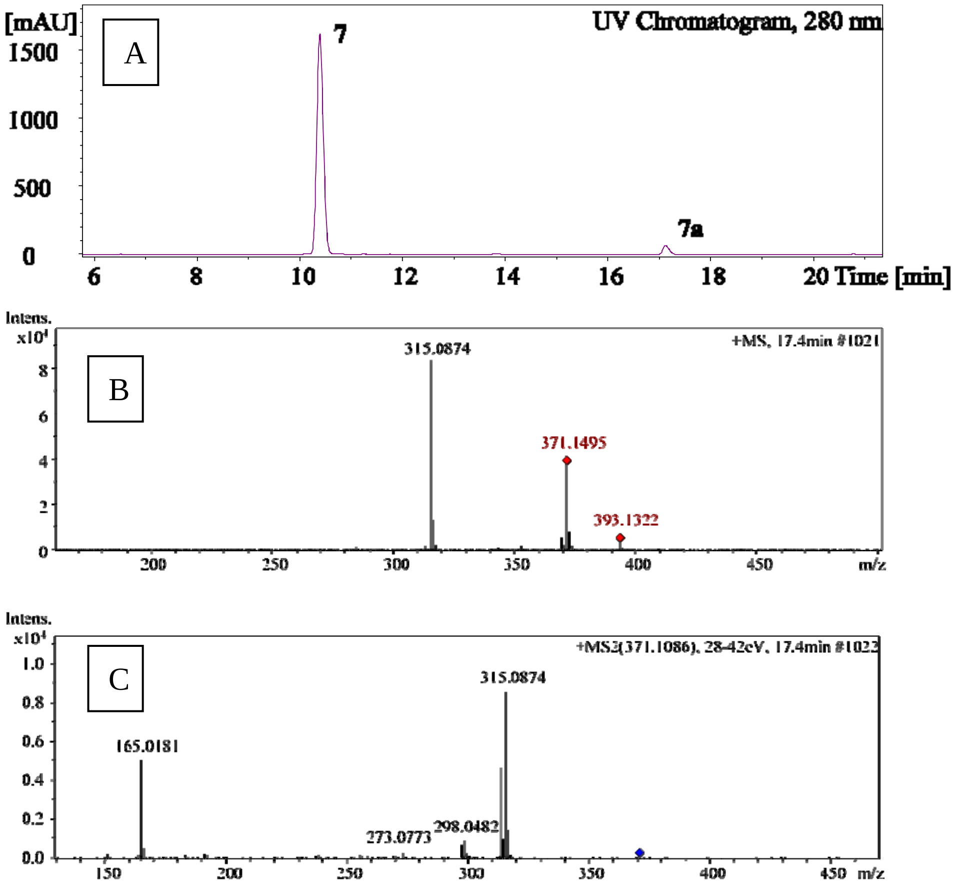

Figure 10S. The prenylated product of hesperitin analysed by HPLC-MS/MS. A, the chromatogram of the prenylated product recorded at 280 nm; B, the parent ion of 6-*C*-prenyl hesperitin at positive mode; C, the daughter ions of 6-*C*-prenyl hesperitin at positive mode.
